# Pneumothorax Revealing the Rupture of a Primary Pleural Hydatid Cyst

**DOI:** 10.7759/cureus.52141

**Published:** 2024-01-11

**Authors:** Chaynez Rachid, Anas Kandil, Mohamed Ijim, Oussama Fikri, Lamyae Amro

**Affiliations:** 1 Pulmonology, Laboratoire de Recherche Morpho Sciences, Faculté de Médecine et de Pharmacie de Marrakech, Université Cadi Ayyad, Centre Hospitalier Universitaire (CHU) Mohammed VI, Hopital Arrazi, Marrakech, MAR

**Keywords:** thoracic surgery, endemic countries, pneumothorax, ecchinococus granulosis, hydatid cyst

## Abstract

Primary pleural hydatid cysts are exceptionally uncommon, even in areas where this parasitic infection is prevalent. The occurrence of pneumothorax can serve as an indicator of this condition, presenting a challenge in both diagnosis and treatment when compared to more common causes of pneumothorax. Moreover, hydatid serology often yields negative results, further complicating the diagnosis. Our case study involves a 15-year-old male who experienced a pneumothorax, subsequently revealing a primary pleural hydatid cyst. This report focuses on elucidating its genesis mechanism and outlining its indicative clinical, radiological, and biological characteristics.

## Introduction

Hydatidosis is a parasitic disease caused by the development of the larvae of *Echinococcus granulosus*. Primary pleural hydatid cyst is a rare yet significant medical condition characterized by the presence of a hydatid cyst within the pleural cavity. Hydatid disease, caused by the parasitic tapeworm *Echinococcus*, typically affects the liver and lungs but can occasionally manifest as cysts within the pleura, the thin membrane surrounding the lungs. This condition arises due to the infestation of *Echinococcus *larvae, leading to the formation of fluid-filled cysts within the pleural space. Primary pleural hydatid cysts present unique challenges in diagnosis and management, requiring a comprehensive understanding of their clinical features, imaging modalities, and treatment strategies to ensure effective patient care. This condition, although uncommon, necessitates prompt recognition and appropriate intervention to prevent complications and promote optimal patient outcomes [1]. We report a case of a pleural hydatid cyst complicated by a compressive pneumothorax. The objective is to describe an exceptional complication of pulmonary hydatid cysts through this observation.

## Case presentation

**Figure 1:**
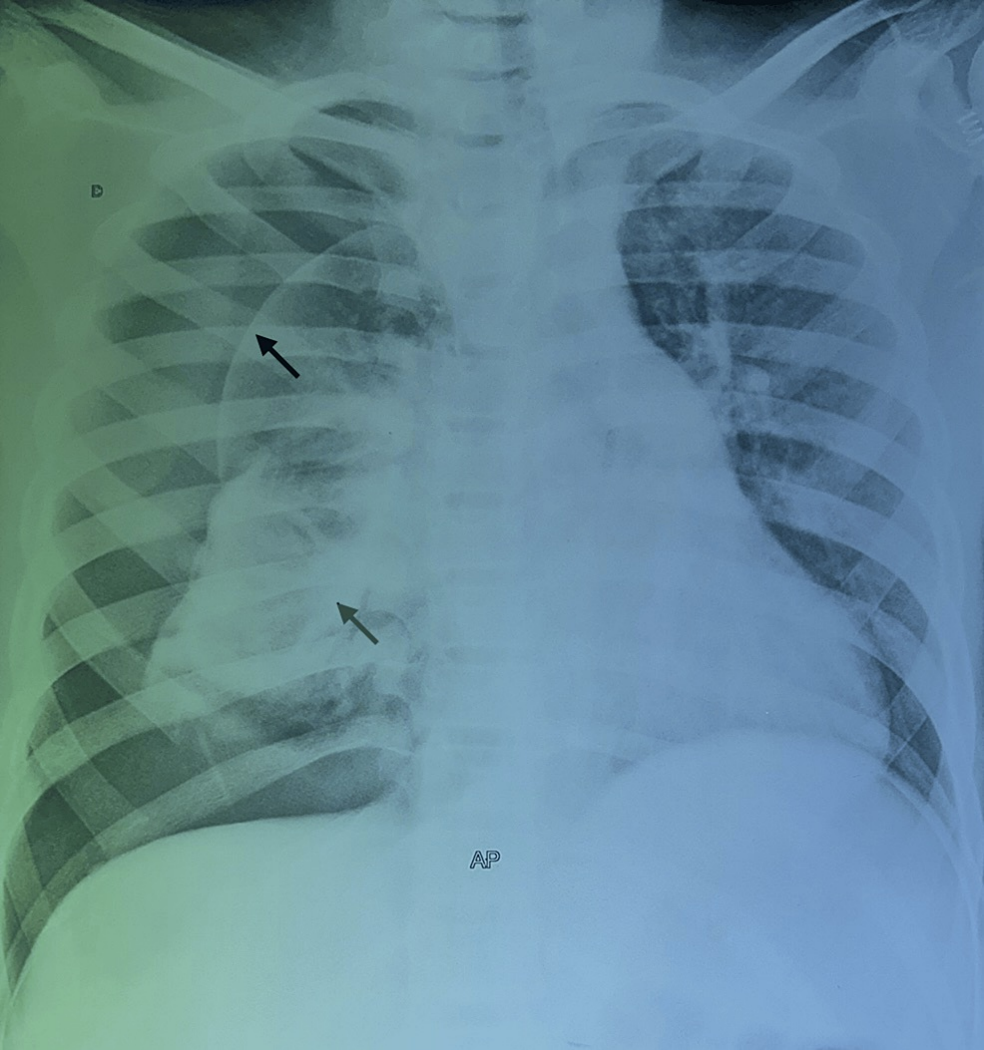
A posteroanterior chest radiograph demonstrates typical features of pneumothorax. The image shows a large pneumothorax with a collapsed lung occupying the entire right hemichamps, communicating with the hydroaeric image and the deviation of the elements of the mediastinum.

The patient was a 15-year-old student living in a rural area and had contact with dogs. The clinical symptomatology dates back to one month, with the progressive onset of a bronchial syndrome consisting of a cough with yellowish sputum and an episode of hydatidoptysis associated with chest pain and dyspnea, all evolving in the context of feverish sensations, night sweats, and a general decline in condition. A physical examination revealed an air effusion syndrome in the right hemithorax. A frontal chest X-ray revealed a large pneumothorax with a collapsed lung. Within this hyperclarity was an excavation with a hydroaeric level (Figure 1).

**Figure 2:**
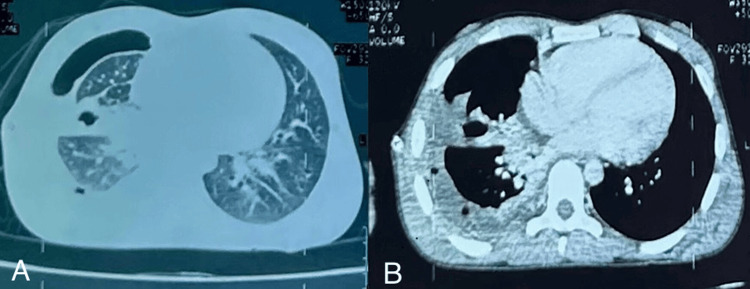
The chest CT scan in the parenchymal (A) and mediastinal windows (B) shows hydropneumothorax. The CT scan image confirmed a right pleural and scissural hydropneumothorax of moderate abundance appearing to communicate with a hydroaeric image in the parenchymal window (A) associated with a pericardial effusion, right laterotracheal and hilar adenopathies measuring 15 mm, and the presence of air bubbles in the pleural cavity in the mediastinal window (B).

Thoracic CT revealed a right pleural and scissural hydropneumothorax of moderate abundance, appearing to communicate with a hydroaeric image, a pericardial effusion, and right laterotracheal and hilar adenopathies measuring 15 mm (Figure 2).

There were no predominantly abdominal localizations. Blood count showed anemia at 8.5 g/dl; C-reactive protein (CRP) was 165 mg/l; liver and kidney function were normal. Hydatid serology was positive at 6.6.

**Figure 3:**
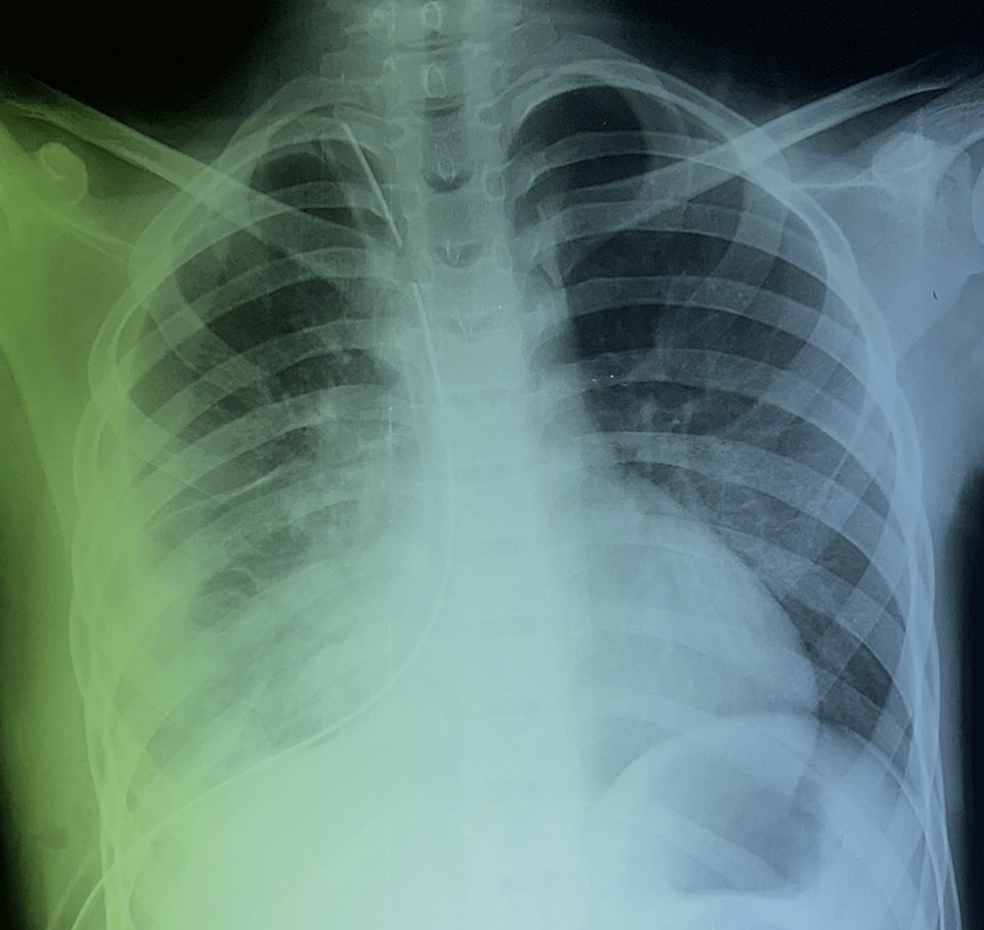
Posteroanterior chest radiograph after pleural decortication The image shows the return of the lung to the wall; mediastinal elements and drains are in place.

Treatment consisted of thoracotomy combined with medical therapy. A posterolateral right thoracotomy was performed, revealing a healthy hydatid cyst at the pleural level with fibrin deposits. The cyst was extracted in its entirety, and the fibrin deposits were removed. Pleural decortication was then performed, and the chest wall was closed after cleaning the large pleural cavity. The postoperative course was straightforward, with the lung parenchyma returning to the wall (Figure 3).

The postoperative course was marked by good clinical, biological, and radiological progress.

## Discussion

The hydatid cyst remains a parasitic disease prevalent in various global regions, particularly around the Mediterranean. While the liver remains the primary site (accounting for 75% of cases), the lungs follow as the second most common site (20% to 40%). Uncommonly, the cysts may occur in the pleural area, representing a mere 1.3% of thoracic occurrences. This affection primarily impacts young adult males [2]. We documented a case involving a young patient who was 15 years old.

Following human infestation with taenia eggs, larval release, facilitated by gastric and enteric digestion, occurs. These larvae traverse the intestinal wall, accessing the bloodstream and settling in organs where they develop into small cysts, growing approximately 2 cm to 3 cm annually. Although commonly found in the liver and lungs, these cysts infrequently manifest in intrathoracic and extrapulmonary regions like the pleura, diaphragm, mediastinum, pericardium, and chest wall. Pleural hydatid cysts can result from the rupture of liver or lung cysts into the pleural space, leading to complications like pneumothorax, pleural effusion, or empyema. The movement of eggs into the lungs during respiration leads to the development of scolexes in a humid environment. These scolexes traverse the alveolocapillary membrane, joining the systemic circulation through the pulmonary veins. While some scolexes settle in organs like the heart, bone marrow, eye, and brain, others migrate into the pleural space due to negative pleural pressure, contributing to the disease's onset in this area. This likely occurred in the presented patients. Within the pleura, cysts position themselves between the parietal pleura and the endothoracic fascia, potentially involving systemic or lymphatic systems. The avascular nature of pleural layers allows for the growth of a hydatid cyst, facilitated by the permeability of the laminated cyst membrane to various nutrients and substances, which traverse the membrane via diffusion. Active transport mechanisms might also play a role [3].

Clinical symptoms are generally nonspecific and poor, resembling other pleural-pulmonary diseases such as chest pain, dyspnea, and dry cough. Diagnosis might occur acutely following a cyst rupture, presenting sudden thoracic pain and dyspnea. Alternatively, the condition can remain asymptomatic for extended periods, with tardy diagnoses. Rarely, signs of mediastinal compression might manifest depending on the cyst's location. Incidental discovery through chest X-rays is also possible. Our patient had subtle clinical signs, as described in the literature. Imaging plays a pivotal role in confirming the diagnosis. Chest X-rays often reveal a distinct, well-defined, homogenous pleural opacity with a water-like appearance. Peripheral calcifications, although rare, may aid in diagnosis. In some instances, characteristic radiographic findings, such as a cystic formation with a calcified wall, strongly suggest the diagnosis [4]. Our patient's chest X-ray exhibited these distinct features, supporting a highly probable diagnosis. Additional investigations aim to precisely locate the cyst (whether cardiac or pleural). Computed tomography verifies the pleural localization of a well-defined, unaltered fluid mass without contrast enhancement, offering higher sensitivity and specificity in diagnosis. An MRI serves as supportive evidence when cysts lack characteristic features on ultrasound or CT, particularly in pseudo-tumoral forms. It precisely delineates cyst topography and relationships with neighboring organs through multiplanar sections. Specific T2-weighted sequences may reveal daughter vesicles or a floating membrane, favoring the diagnosis, alongside a peripheral T1- and T2-hypointense signal indicative of calcium deposits, pathognomonic in diagnosis [5]. Biochemically, hypereosinophilia is typically absent in intrathoracic hydatid disease.

Immunological tests like the IgG enzyme-linked immunosorbent assay (ELISA), indirect hemagglutination, and Western blot assay may aid but possess only around 60% sensitivity. Combining two or more biological tests with radiological imaging enhances diagnostic accuracy. Immunology sensitivity notably increases in cases of complications or associated liver cysts. Our patient had positive immunologic tests, consistent with literature findings. In the absence of cyst rupture, puncturing the cyst is strictly contraindicated, often leading to a cytological or pathological diagnosis post surgical excision [6]. The rarity of this condition and its lack of clinical, radiological, and biological specificity make a positive diagnosis challenging. Multiple other causes of cystic lesions must be considered, such as bronchogenic cysts, enteric cysts, pleuropericardial cysts, thymic cysts, and lymphangiomas [7]. Definitive diagnosis often relies on visualizing the hydatid membrane, daughter vesicles, or pathological examination of the operative specimen in cases of infected or thickened cysts. Mobilization of daughter vesicles is regarded as a sign of secondary pleural hydatidosis, rarely observed in its primary form. In our case, imaging played a critical role, almost confirming the diagnosis preoperatively. Surgical intervention and subsequent parasitological examination validated the previously obtained imaging data and established a definitive diagnosis [8].

## Conclusions

Pleural hydatidosis is a possible cause of pneumothorax and should not be overlooked in endemic areas. The pleural hydatid cyst is a rare localization of extrathoracic hydatid disease, and although not frequent in Morocco, the diagnosis should be discussed in the presence of a pneumothorax associated with a hydroaeric image. The treatment is essentially surgical. Prevention remains the best way to reduce the incidence of this pathology.
